# Gut microbiota composition and functional prediction in diarrhea-predominant irritable bowel syndrome

**DOI:** 10.1186/s12876-021-01693-w

**Published:** 2021-03-05

**Authors:** Lijun Mei, Jiaoli Zhou, Yimo Su, Kunhong Mao, Jing Wu, Caicai Zhu, Ling He, Ying Cui

**Affiliations:** 1 Jiangxi University of Traditional Chinese Medicine Affiliated Hospital, Nanchang, 330006 China; 2 Jiangxi University of Traditional Chinese Medicine, Nanchang, 330004 China; 3 Jiangxi Maternal and Child Health Hospital, Nanchang, 330006 China

**Keywords:** Diarrhea-predominant irritable bowel syndrome, Gut microbiota, Functional prediction

## Abstract

**Background:**

Irritable bowel syndrome (IBS) is common and difficult to treat and its pathogenesis is closely related to gut microbiota. However, differences in gut microbiota of patients in different regions make it more difficult to elucidate the mechanism of IBS. We performed an analysis of gut microbiota composition and functional prediction in Chinese patients with diarrhea-predominant IBS (IBS-D).

**Methods:**

Fecal samples were obtained from 30 IBS-D patients and 30 healthy controls (HCs) in Nanchang, China. Using 16S gene sequence profiles, we analyzed the abundance of dominant microbiota at different taxonomy levels. Based on 16S information, Phylogenetic Investigation of Communities by Reconstruction of Unobserved States (PICRUSt) was used to predicting the function of gut microbiota.

**Results:**

Compared to HCs, gut microbiota richness but not diversity was decreased in IBS-D patients. The abundant phyla *Firmicutes*, *Fusobacteria* and *Actinobacteria* decreased significantly, and *Proteobacteria* increased significantly in IBS-D patients. PICRUSt indicated that function expression of gut microbiota in IBS-D patients was up-regulated in metabolism of cofactors and vitamins, xenobiotics biodegradation and metabolism, and down-regulated in environmental adaptation, cell growth and death.

**Conclusions:**

Compared with the normal population in China, IBS-D patients are characterized by complex and unstable gut microbiota, which may influence inflammation and metabolism of the host.

**Supplementary Information:**

The online version contains supplementary material available at 10.1186/s12876-021-01693-w.

## Background

Irritable bowel syndrome (IBS) is a common functional gastrointestinal (GI) disorder that affects 7–21% of the population worldwide [1] and 5–10% in most European countries, the US and China [2], but it differs depending on regions and diagnostic criterion. It causes significant socioeconomic burden on society [3]. Based on the predominant stool pattern, patients with IBS are categorized four subtypes [4]: constipation-predominant IBS (IBS-C), diarrhea-predominant IBS (IBS-D), mixed IBS (IBS-M), and unclassified IBS (IBS-U).

The pathophysiology of IBS has remained elusive and its linked factors include abnormalities of GI motility, visceral hypersensitivity, post-infectious low-grade inflammation, alteration of gut microbiota, brain-gut interactions and genetic factors [5–10]. Interestingly, one of our early studies showed abnormalities in salivary amylase in patients with IBS, suggesting autonomic dysfunction caused by psychological factors may be part of pathogenesis [11].

The past decade has seen considerable studies on gut microbiota due to its key role in human diseases, especially for IBS [12]. Previous studies have found a large number of different kinds of intestinal flora in the human gut, but most of them were limited to the number and abundance of bacteria, which are only the tip of the iceberg compared to the function of these bacteria or the whole bacteria of nature. In addition, gut microbiota represents one source of human genetic and metabolic diversity and differ among human populations [13]. The 5th International Meeting on Inflammatory Bowel Diseases (IBD) pointed out that the increased incidence of IBD among migrants from low-incidence to high-incidence areas within the same generation suggests a strong environmental influence, and added that the importance of gut flora in intestinal homeostasis and inflammation must be reinforced [14]. Therefore, more gut microbiota data of IBS patients from different regions are required.

With the development of next-generation high-throughput sequencing, the investigation of the human gut microbiota has been ever more feasible. Furthermore, bacterial functional profiles can be predicted by inferring the metagenome of the closest available whole genome sequences using 16S gene sequence profiles. Based on 16S information, Phylogenetic Investigation of Communities by Reconstruction of Unobserved States (PICRUSt) recaptures key findings from the Human Microbiome Project and accurately predicts the abundance of gene families in host-associated and environmental communities, with quantifiable uncertainty [15]. Using PICRUSt, several differences in such variables that may be of pathophysiological significance, but the findings are challenging to interpret and should be considered with caution [16]. A systematic review of gut microbiota in patients with IBS based on 16S sequencing showed that alterations of gut microbiota exist in patients with IBS and have a significant association with the development of IBS [17]. However, data on specific bacterial groups in IBS are conflicting and still inconclusive according to another systematic review [18].

Through a case–control study, we aimed to address three questions related to gut microbiota in IBS-D patients and health population of Nanchang, China: What are the differences in the composition and abundance of gut microbiota between the two populations? In what ways does the function of these altered bacteria affect the host? What are the probable causes of these differences?

## Methods

### Study design

This study included 30 patients with IBS-D and 30 healthy controls (HCs), aged from 20 to 64 and 24 to 65 years, respectively. Inclusion criteria met the Rome IV diagnostic criteria [19] for IBS-D. Gender, age, BMI and staple food of all participants were collected, which were shown in Table 1 (Details can be found in Additional file 1: Table S1). All the participants lived in Nanchang. Exclusion criteria for all subjects included: (1) taking antibiotics, probiotics, or other treatments, within 4 weeks; (2) inflammatory bowel disease, peptic ulcer, diverticulitis or infectious gastroenteritis; (3) pregnant, menstruating and lactating women; (4) any psychiatric comorbidity; (5) excessive physical exercise.

**Table 1:** Characteristics and OTUs of two groups

Group	IBS-D	HCs	*P* value
Number	30	30	–
Gender			
Male	13	15	0.293
Female	17	15	
Age	40.3 ± 14.7	40.9 ± 14.4	0.874
BMI	19.97 ± 5.58	21.39 ± 3.83	0.014
Staple food			
Rice	26	27	1.000
Wheat	4	3	
OTUs	1547	1388	0.001

### Sample collection and DNA extraction

A single fecal sample was collected by each participant at home, and immediately stored at – 20 ℃, then transferred to – 80 ℃ for longer-term storage. Fecal bacteria genomic DNA was extracted with cetyltrimethyl ammonium bromide (CTAB).

### 16S rDNA gene sequencing

The V4 hypervariable region of the 16S rDNA gene was amplified using Phusion ® High-fidelity PCR Master Mis with GC Buffer (New England Biolabs, US). All PCR products were visualized on agarose gels (2% in TAE buffer), and purified with a DNA gel extraction kit (Qiagen, Germany). Paired-End (PE) amplicon library was constructed using a TruSeq® DNA PCR-Free Sample Preparaion Kit (Illumina, US) and quantified by Qubit, then sequencing was performed using the Illumina Hiseq platform (APTBIO Technology, Shanghai, China).

### Data and bioinformatics analysis

FLASH and Trimmomatic software were used to splice and filter the raw tags, and the effective tags were obtained by comparing in the Gold database. The effective tags were clustered with Uparse software, and the sequences were clustered into operational taxonomic units (OTUs) with 97% consistency. The representative OTUs were selected for species annotation analysis with the method of Mothur and SILVA's SSUrRNA database (The threshold value was set at 0.8–1).

Qiime (Version 1.9.1) and R (Version 2.15.3) were used for data analysis. The indexes, including Observed-species, Chao1, Shannon, Simpson and ACE, were used to describe the Alpha-diversity. By calculating the Unifrac distance, constructing the unweighted pair-group method with arithmetic mean (UPGMA) sample cluster tree and drawing principal component analysis (PCA), principal co-ordinates analysis (PCoA) and non-metric multi-dimensional scaling (NMDS) diagrams, the Beta-diversity was carried out. Based on KEGG database, PICRUSt analysis was applied to predict the functional profiling of microbial communities according to 16S sequencing data. T-test and Wilcox test were used for statistical analysis. T-test test and LDA Effect Size (LEfSe) analysis were used for the analysis of different species between groups. LEfSe analysis using the LEfSe software, the LDA Score screening value is 4.

## Results

### Sequencing quality analysis

A total of 3,667,351 valid tags sequences were generated from 60 samples, and the average number of high-quality sequences obtained per sample was 61,132. The average length of valid Tags sequences of all samples was between 406 and 421 bp, with an average of 414 bp. Sample sequencing depth was between 99 and 100%, and all samples were fully sequenced.

### The number of OTUs

The number of common OTUs between the patients of IBS-D and HCs was 813. Meanwhile, the number of proper OTUs in the patients of IBS-D was 734, and that in HCs was 575. Details can be found in Additional file 2: Table S2.

### Characterization of fecal microbiota

**Fig. 1:**
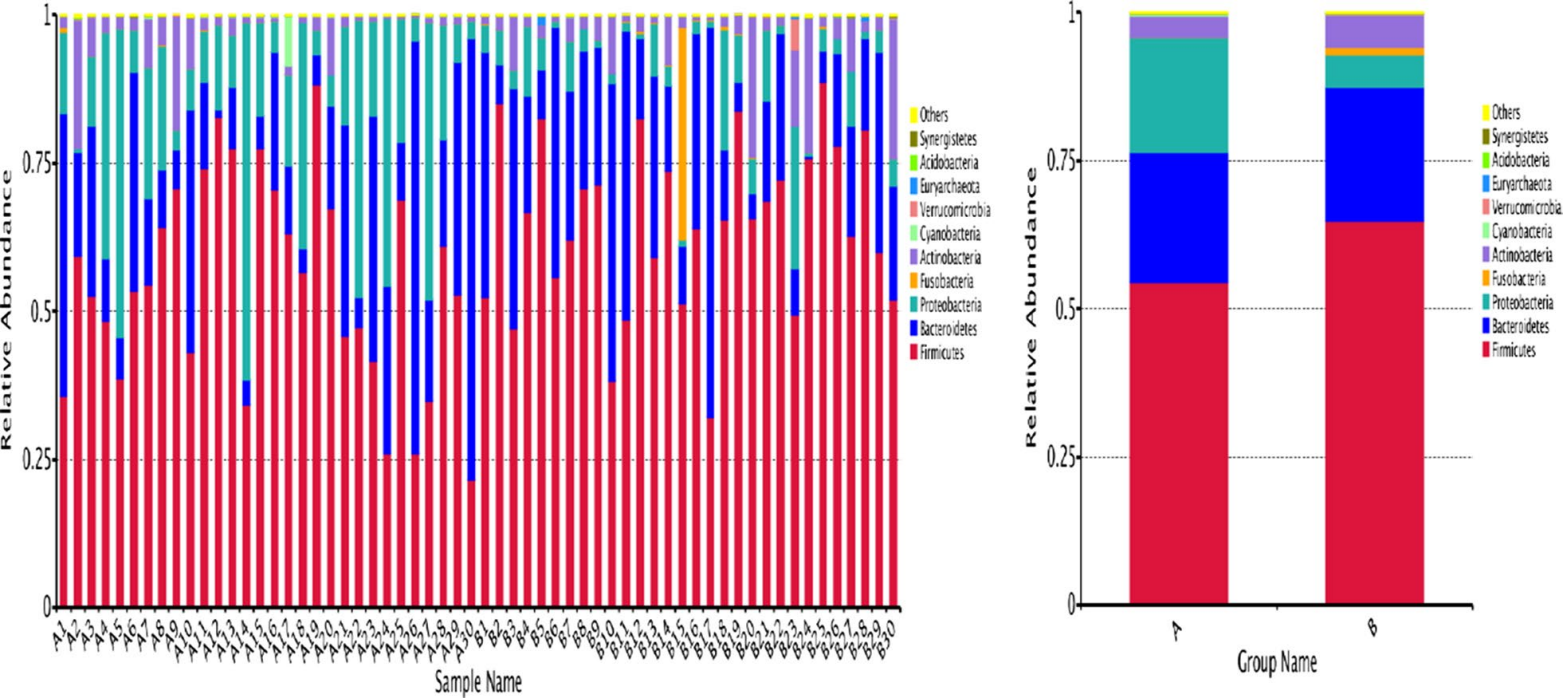
Top10 histogram of relative abundance of species (phylum level): **a** IBS-D, **b** HCs. The abscissa is the name of the sample, the ordinate represents the relative abundance, and the others represent the sum of the relative abundance of all the other phylum except the top10 in the figure

There is significant difference in gut microbiota composition between IBS-D patients and HCs, details can be found in Additional file 3: Table S3, Additional file 4: Table S4.The phylum level was taken as an example to show a histogram of relative abundance of species. As shown in Fig. 1, we found that compared with HCs, *Firmicutes* (*P* < 0.05), *Fusobacteria* (*P* < 0.01), *Actinobacteria* (*P* < 0.01) decreased significantly, and *Proteobacteria* increased significantly (*P* < 0.01) in IBS-D patients.

**Fig. 2:**
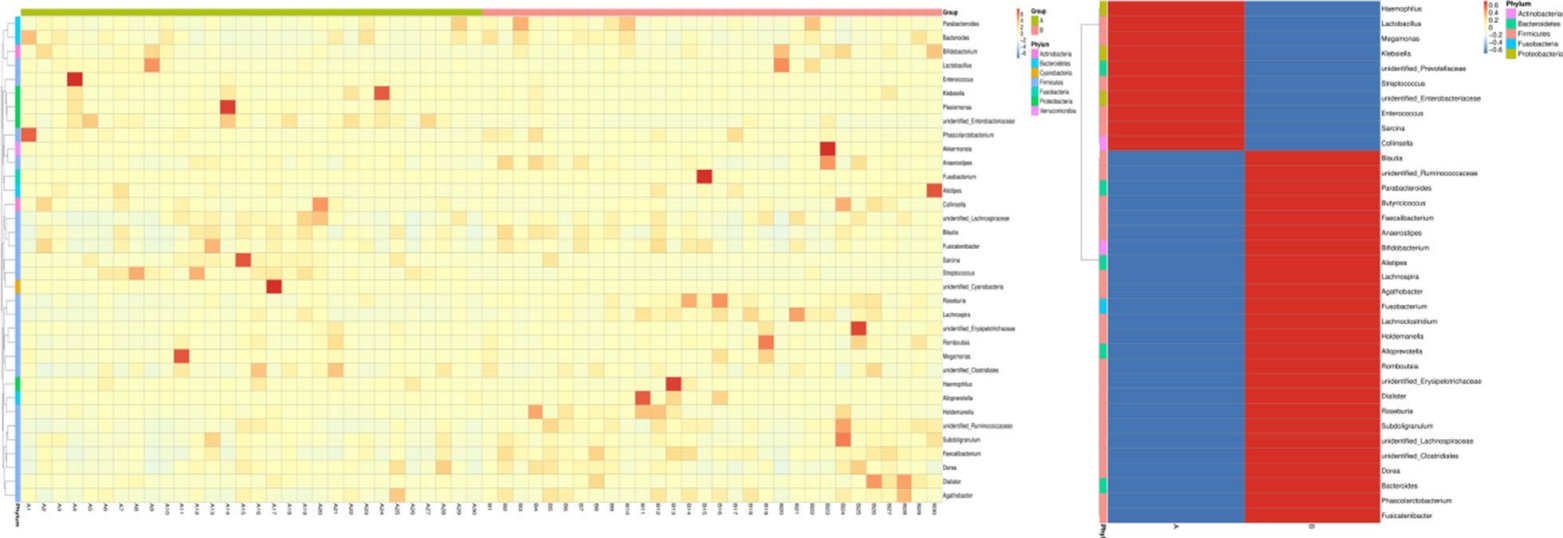
Top35 heat map of species abundance clustering: longitudinal are the sample information, horizontal are the species annotation information. The cluster tree on the left is a species cluster tree, and the contrast between the two groups is on the right. The corresponding value of the heat map is the Z value of the relative abundance of species in each row after the normalization treatment

The genus level was taken as an example to show the heat map of species abundance clustering. The results were shown in Fig. 2 that compared with HCs, *Enterobacteriaceae* significantly increased (*P* < 0.01), and *Alloprevotella* (*P* < 0.01), *Fusobacterium* (*P* < 0.01) significantly decreased in IBS-D patients.

**Fig. 3:**
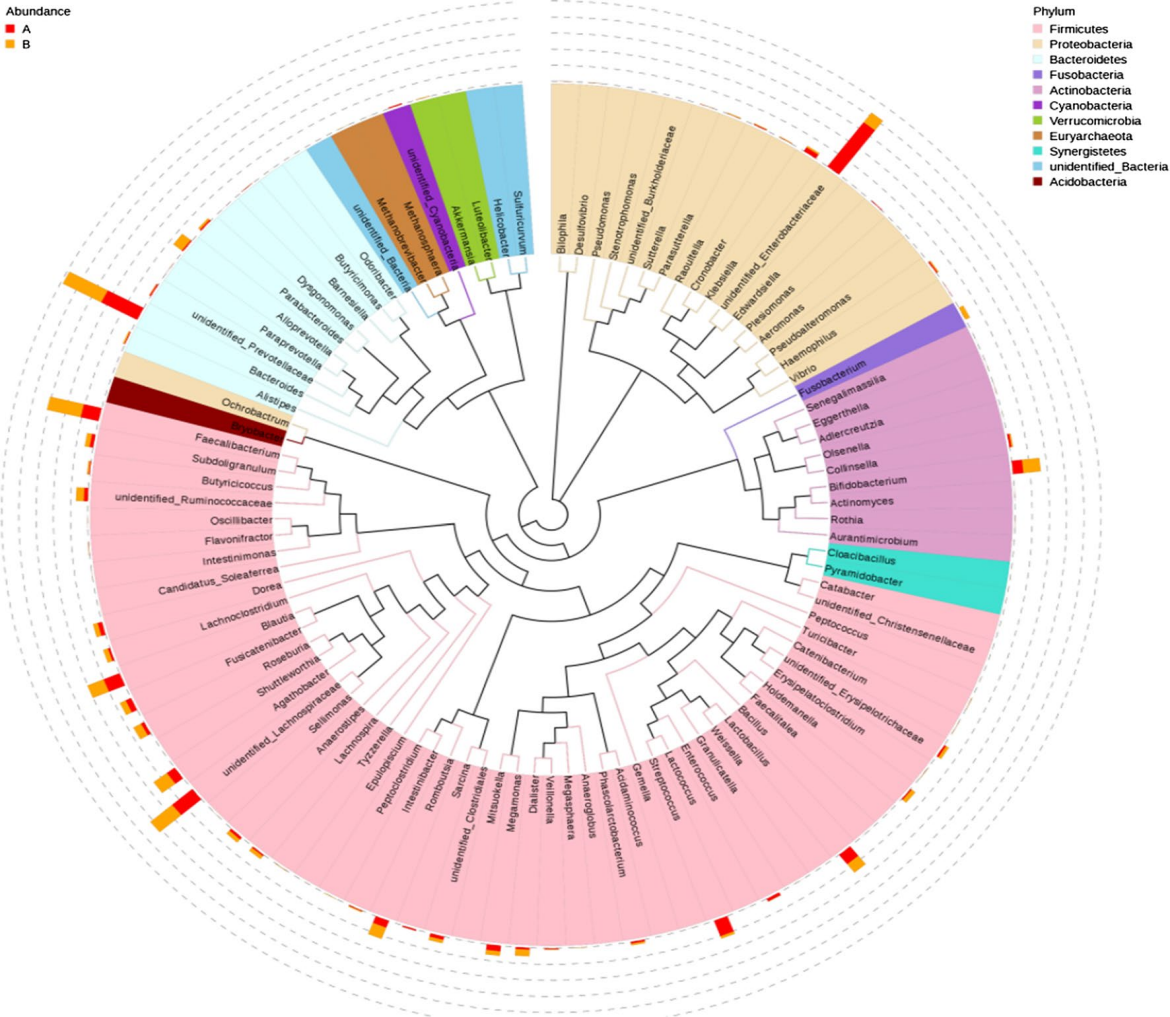
Phylogenetic relationships in genus level: the colors of the branches and fan-shaped segments represent their corresponding phylum, and the pileup column on the outside of the fan ring represents the abundance distribution information of the genus in different samples

In order to further study the phylogenetic relationship of genus level species, the representative sequences of top100 genus were obtained by multi-sequence alignment and shown in Fig. 3.

**Fig. 4:**
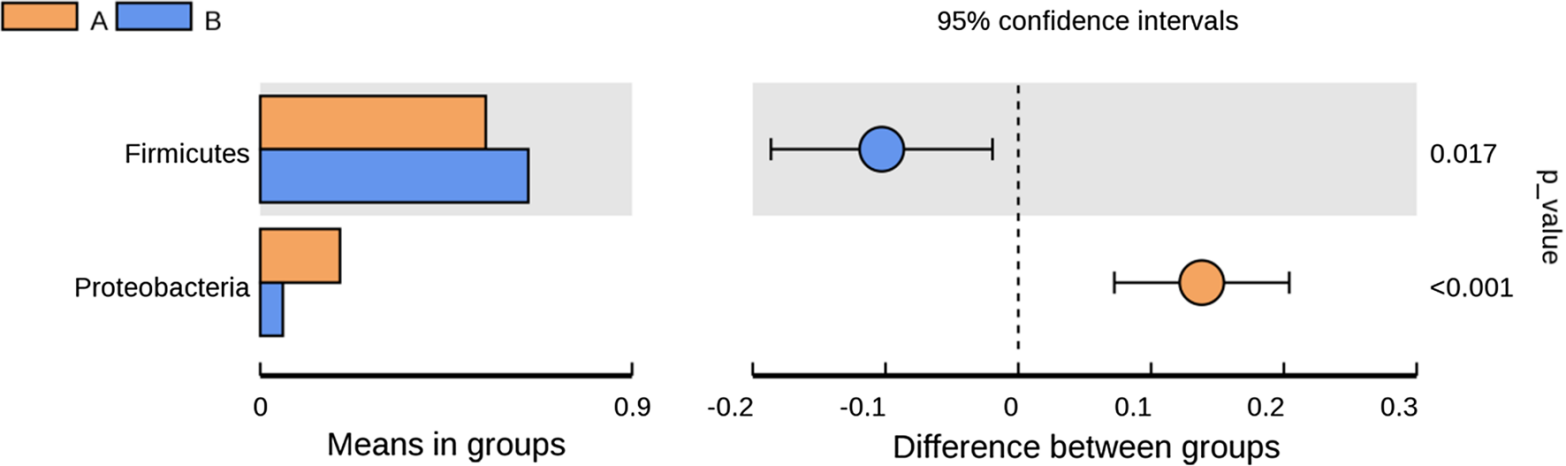
Analysis of species differences between groups

Statistical analysis was performed to find species with significant differences between the two groups. Using T-test, the phylum level was taken as an example to show the difference of species between the two groups which can be seen in Fig. 4. The result showed that the species that differed significantly between the two groups were *Firmicutes* and *Proteobacteria*.

**Fig. 5:**
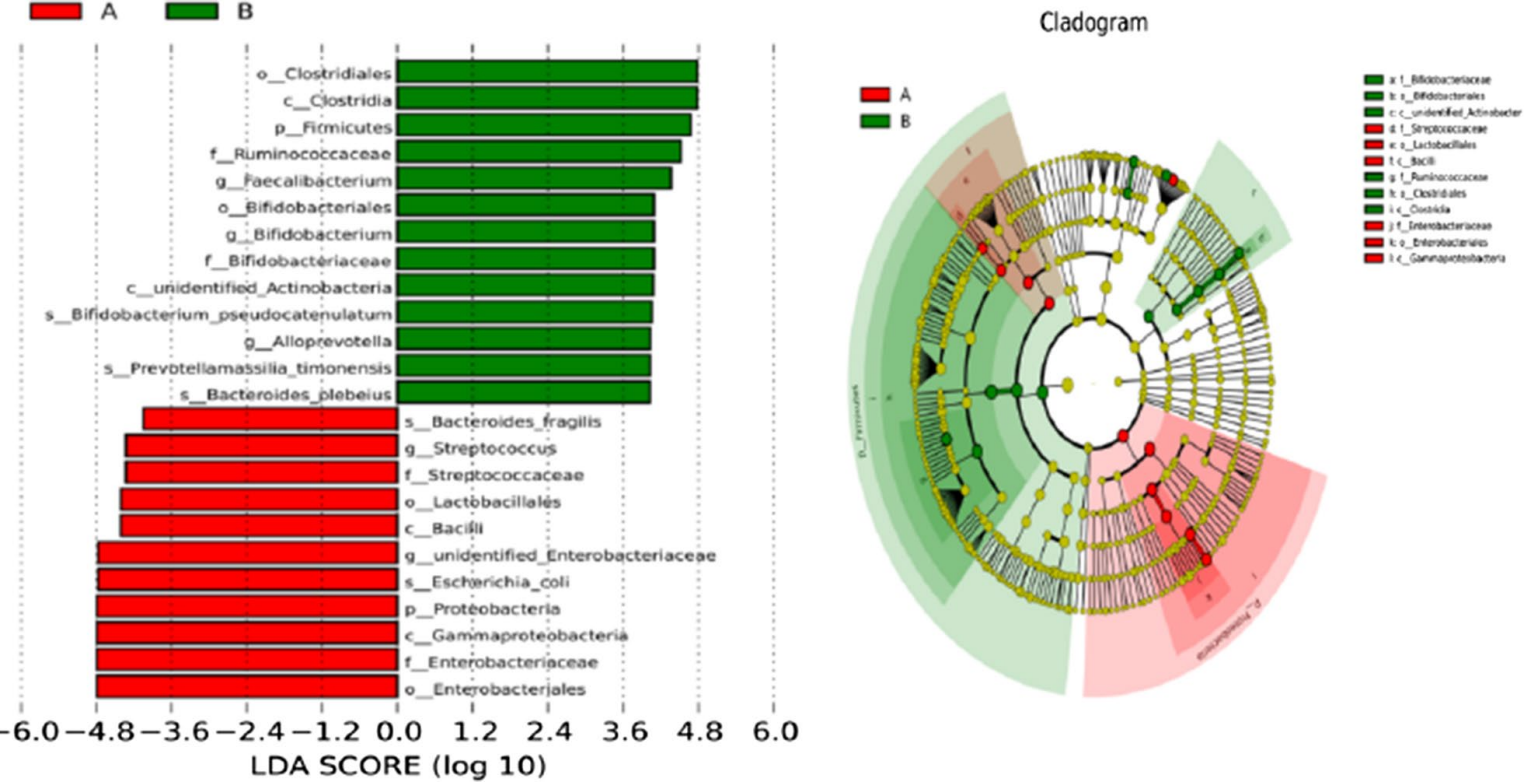
Distribution histogram of LDA score and cladogram of species: **a** IBS-D, **b** HCs. The length of the bar chart on the left represents the influence of different species. On the right, the circles radiating from the inside out represent taxonomic levels from phylum to genus (or species); each small circle at a different classification level represents a classification at that level, and the diameter of the small circle is proportional to the relative abundance. Coloring principle: red is the IBS-D, green is HCs, and yellow is the species with no significant difference

In order to emphasize the statistical significance and biological correlation, we performed LEfSe analysis on two groups of bacteria. Using LEfSe, we can identify the characteristics and related categories of different abundance. As shown in Fig. 5, the biomarker of HCs included *Ebterobacteriales*, *Gammaproteobacteria*, *Proteobacteria*, and those of IBS-D included *Clostridiales*, *Clostridia*, *Firmicutes*.

### Alpha diversity

**Fig. 6:**
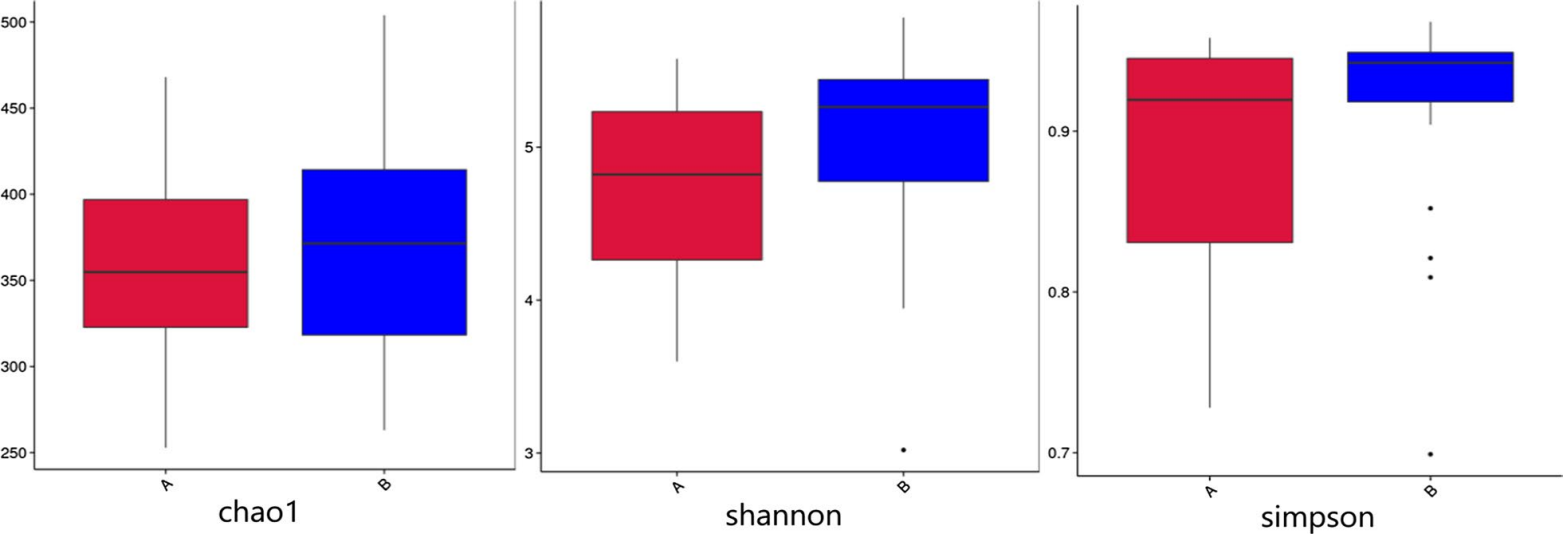
Boxplot of Alpha diversity index between IBS-D and HCs

Alpha diversity index under 97% consistency threshold for different samples (shannon, simpson, chao1, ACE, goods_coverage, PD_whole_tree) was used for statistics (Table 2, data quantity selected during homogenization, cutoff = 48,286). There is a significant difference in community diversity between IBS-D and HCs (*P* < 0.05). The result indicated that community diversity of IBS-D is lower than that of HCs. There is no significant difference in community richness and sequencing depth between IBS-D and HCs. The results of difference analysis between groups with Alpha diversity index were shown in Fig. 6.

**Table 2:** Comparison of Alpha diversity index between IBS-D and HCs

Group	Species	Shannon	Simpson	Chao1	ACE	Goods_coverage	PD_whole_tree
IBS-D	311	4.708	0.890	357.980	374.214	0.998	44.200
HCs	330	5.084	0.921	372.200	385.747	0.999	42.731
*P* value	0.191	0.011	0.191	0.374	0.602	0.445	0.343

### Beta diversity

**Fig. 7:**
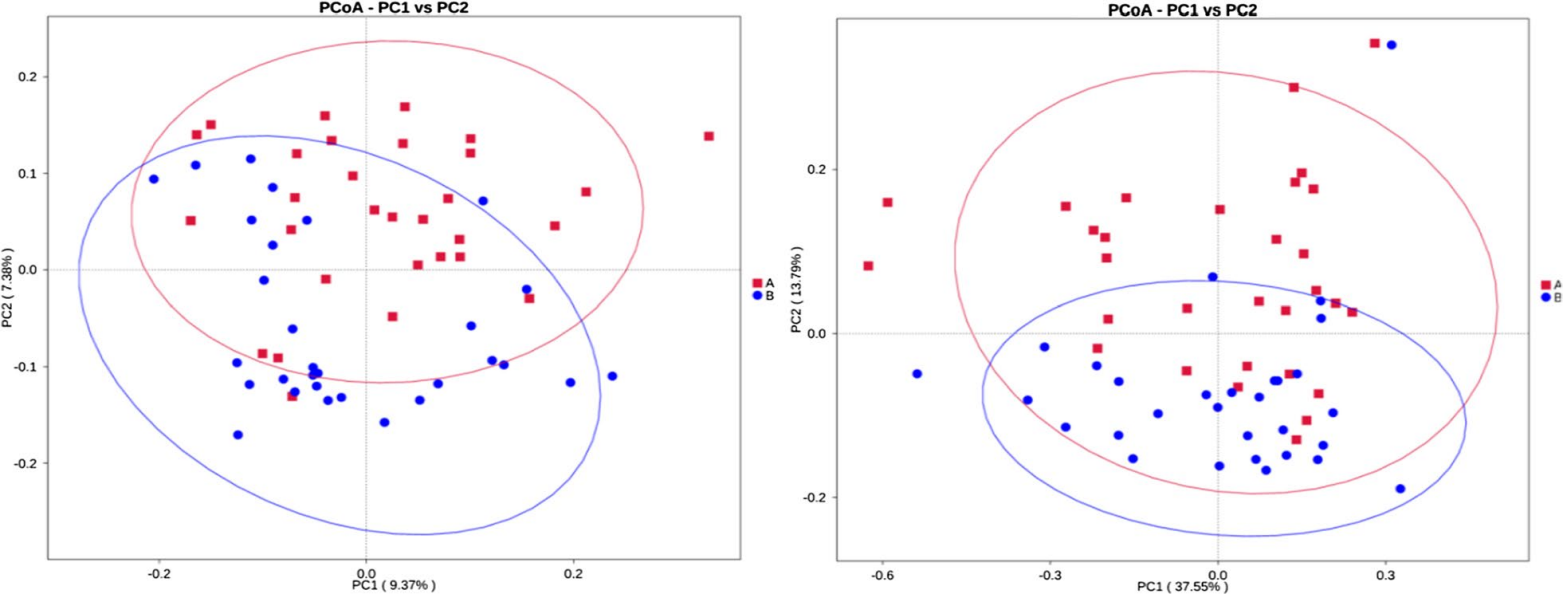
PCoA analysis. **a** IBS-D, **b** HCs. The x-coordinate represents one principal component, the y-coordinate represents another principal component, and the percentage represents the contribution of the principal component to the sample difference. Each point in the diagram represents one sample, and samples from the same group are represented in the same color

PCoA is used to describe the sample distance. We performed PCoA analysis based on weighted unifrac distance and unweighted unifrac distance (Fig. 7). The closer the sample distance is, the more similar the species composition. Details can be found in Additional file 4: Table S4.

**Fig. 8:**
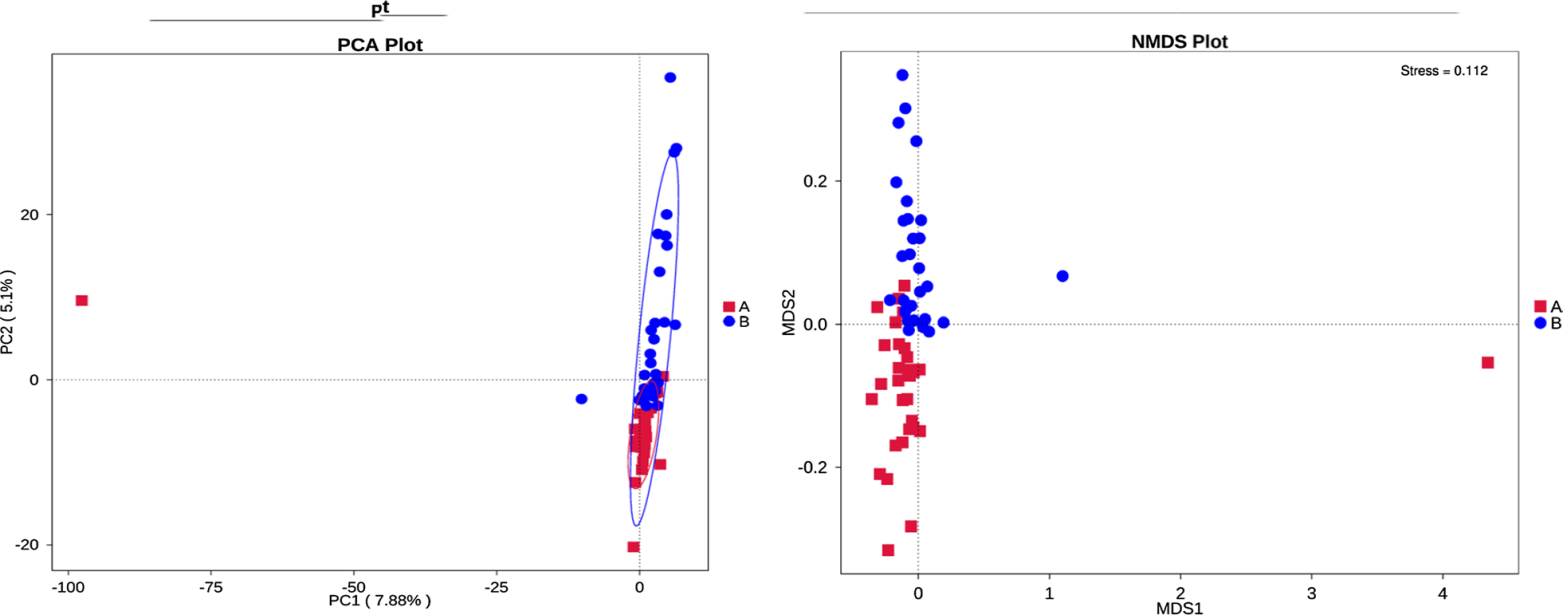
PCA and NMDS analysis: **a** IBS-D, **b** HCs. The left is PCA analysis, the x-coordinate represents the first principal component, the y-coordinate represents the second principal component, and the percentage represents the contribution of the principal component to the sample difference. The right is NMDS analysis

PCA can extract two coordinate axes that reflect the differences between samples to the greatest extent, so as to reflect the differences between multidimensional data on the two-dimensional coordinate diagram, and then reveal the simple rules in the background of complex data. The more similar the community composition of the samples, the closer they were in the PCA (Fig. 8). In order to overcome the shortcomings of linear models (including PCA and PCoA) and better reflect the nonlinear structure of ecological data, we also conducted NMDS analysis (Fig. 8).

**Fig. 9:**
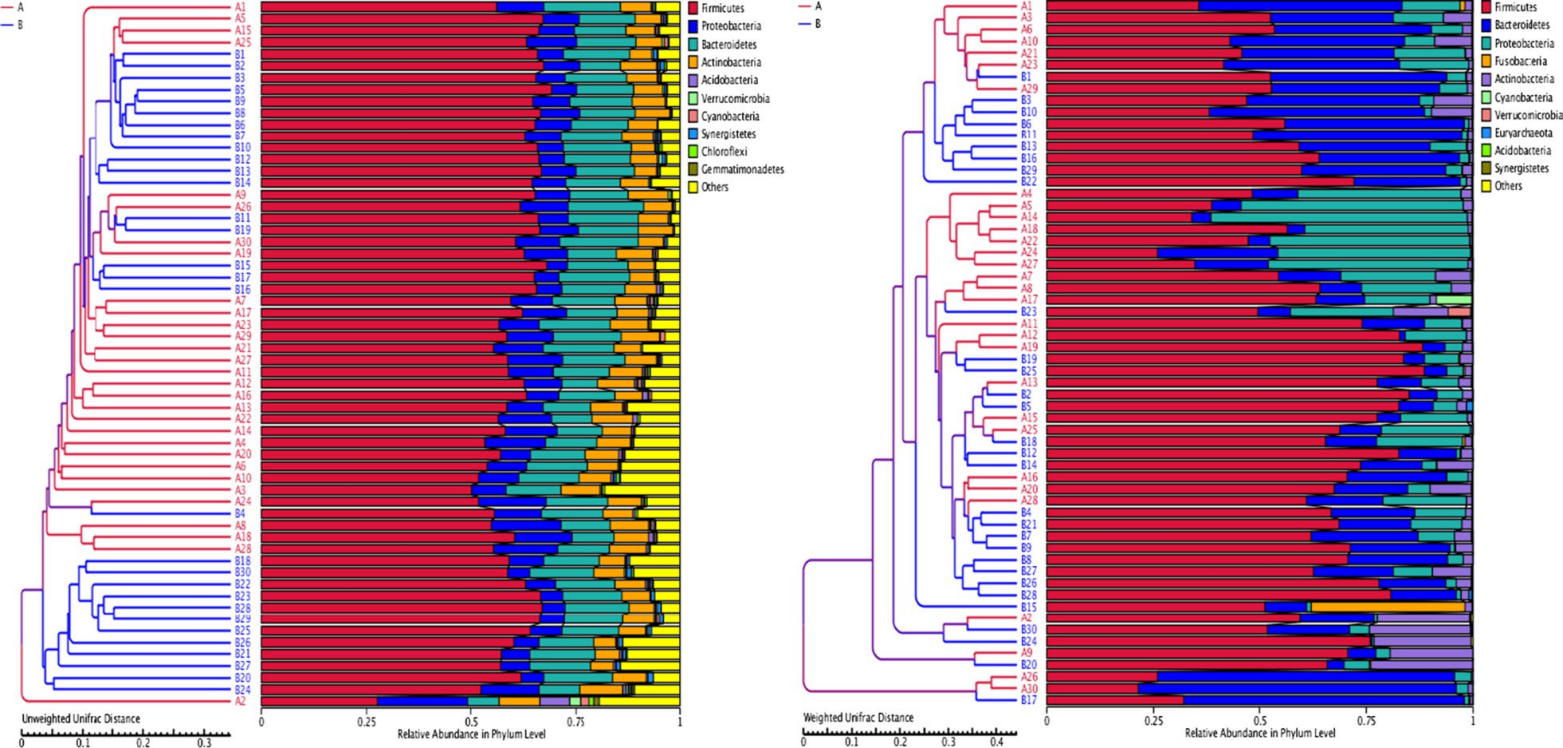
Clustering tree of UPGMA: Left image is based on unweighted unifrac distance, and right image is based on weighted unifrac distance

In order to study the similarity between different samples, we constructed a cluster tree of samples by cluster analysis of samples. Weighted unifrac distance matrix and unweighted unifrac distance matrix were used for UPGMA clustering analysis, and the clustering results were integrated with the relative abundance of species of each sample at the phylum level, as shown in Fig. 9.

### PICRUSt analysis

**Fig. 10:**
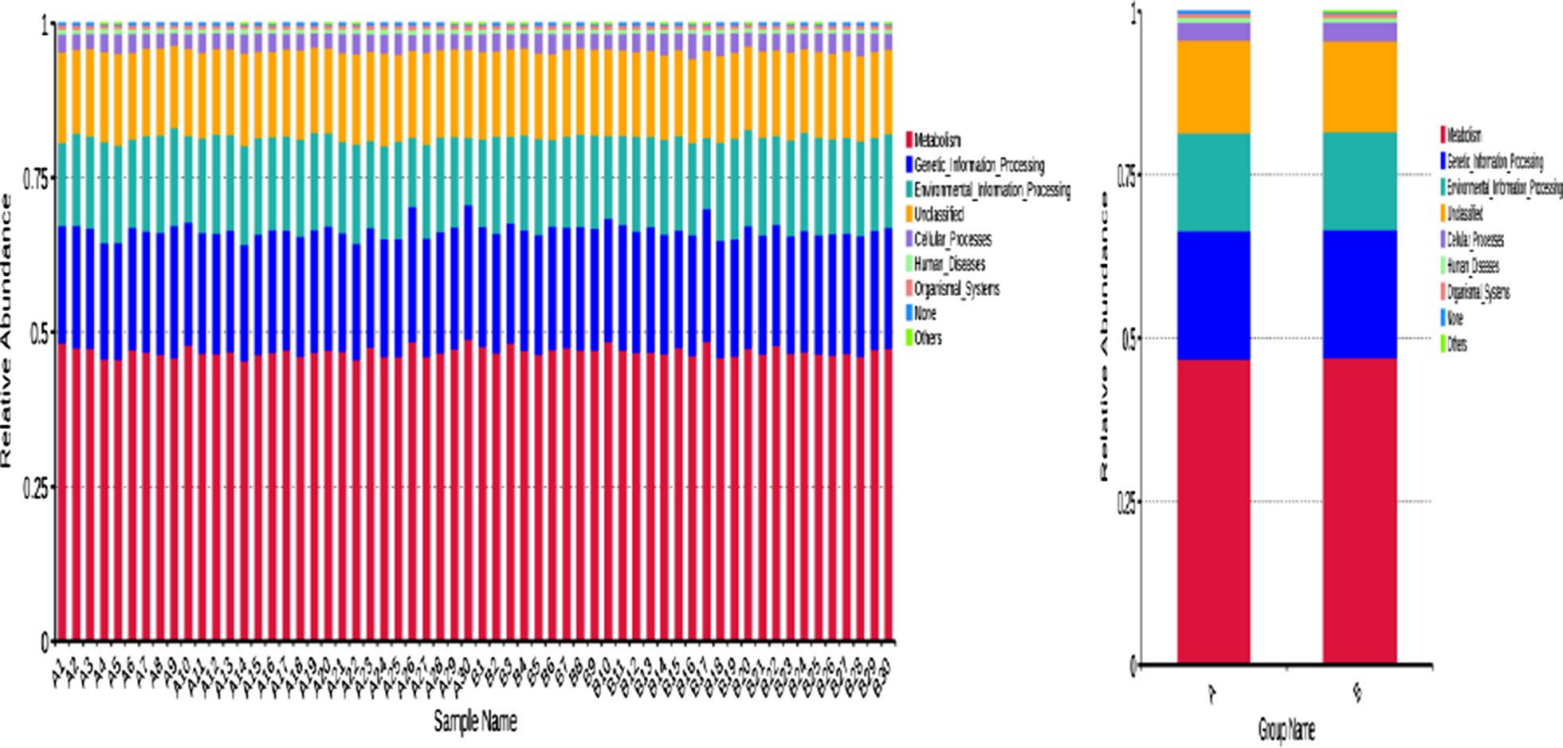
Histogram of functional annotation relative abundance. **a** IBS-D, **b** HCs. The abscissa is the name of the sample, the ordinate represents the relative abundance, and the Others represent the sum of the relative abundance of all the other phylum except the top10 in the figure

**Fig. 11:**
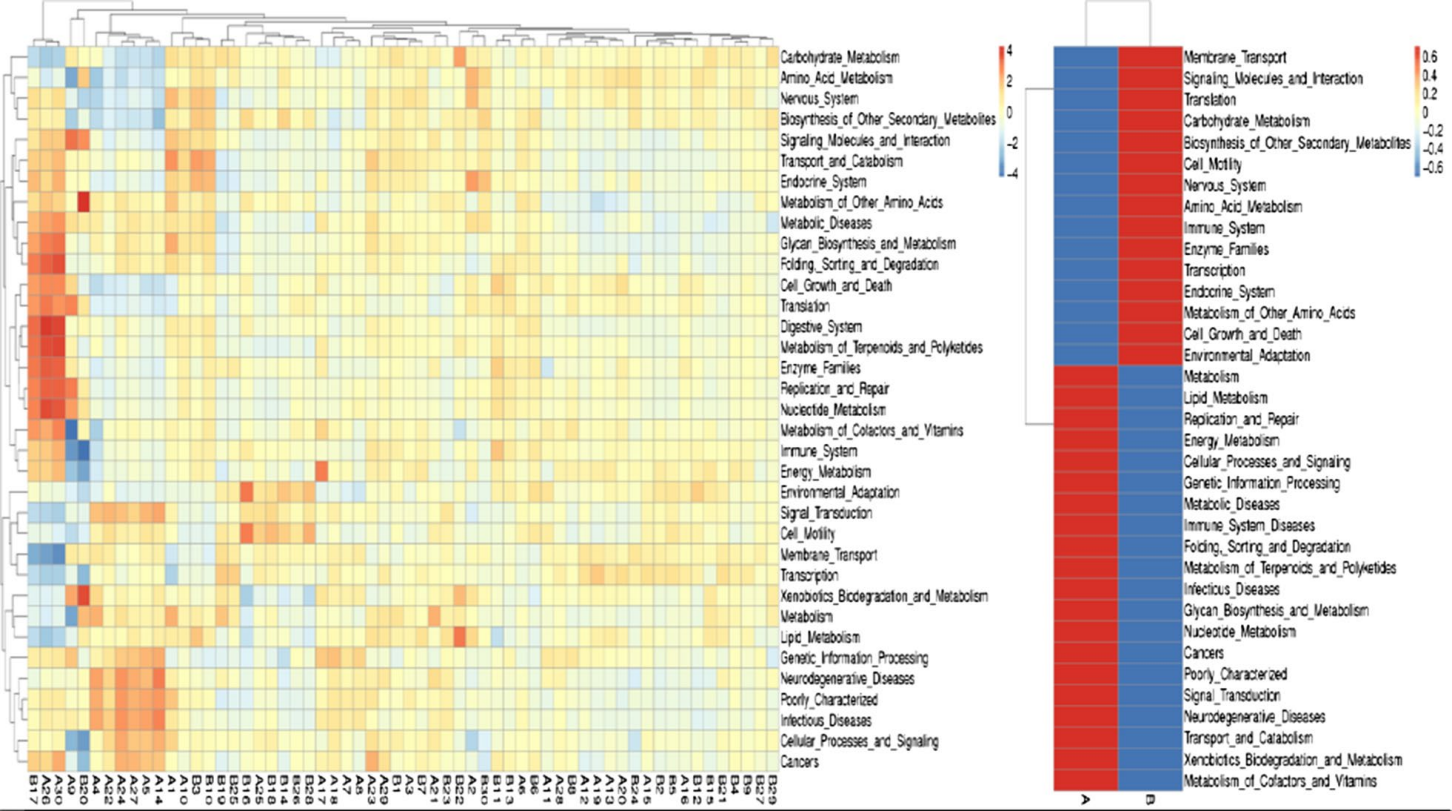
Functional annotation clustering heat map. **a** IBS-D, **b** HCs. The cluster tree on the left is a functional annotation cluster tree, and the contrast between the two groups is on the right

Taking the level 1 as an example, according to the database annotation results, we select the functional information of the top 10 in the maximum abundance of each sample or group, and generate a histogram of the relative abundance of functions, so as to visually view the functions and their proportions with the high relative abundance of each sample. As shown in Fig. 10, the functional genes of the two groups were mainly involved in metabolism, genetic information processing and environmental information processing. According to the functional annotation and abundance information of the samples in the database, the top 35 functions of the abundance and their abundance information in each sample were selected to draw a heat map, and clustering was carried out from the functional difference level. In IBS-D patients, the function expression of gut microbiota was up-regulated in metabolism of cofactors and vitamins, xenobiotics biodegradation and metabolism, and down-regulated in environmental adaptation, cell growth and death, metabolism of other amino acids (Fig. 11). Details can be found in Additional file 5: Table S5, Additional file 6: Table S6, Additional file 7: Table S7, Additional file 8: Table S8.

## Discussion

The analysis of the relationship between gut microbiota and IBS by 16SrDNA sequencing is not new, but this study is the first to analyze the differences of gut microbiota between IBS-D patients and health population in Nanchang, China. Furthermore, we performed PICRUSt analysis to predict the function of gut microbiota.

### The composition of gut microbiota in IBS-D patients was abnormal

In this study, we found that there was significant difference in gut microbiota of IBS-D patients compared to those of HCs in China. At phylum level, *Firmicutes* and *Fusobacteria* decreased significantly, and *Proteobacteria* increased significantly in IBS-D patients. In reviewing the literature, decreased levels of *Firmicutes* and increased levels of *Bacteroidetes* were found in 2 studies [20, 21], one in China, but 4 studies [22–25] reported opposite results, and 3 studies [24–26] found that *Proteobacteria* were increased. In assessing genus level, *Enterobacteriaceae* significantly increased, and *Alloprevotella*, *Fusobacterium* significantly decreased. The results indicated that IBS-D might be related to potential inflammation. Two studies confirmed the hypothesis that *Enterobacteriaceae* increases in IBS-D patients and had significantly lower relative abundance after Rafaximin treatment [27, 28]. Three studies assessing *Bacteroides* demonstrated significant increase in IBS-D patients, whereas 2 studies showed insignificant results compared to controls [21, 29–32]. Moreover, 3 studies assessing genus *Bifidobacterium* showed a significant decrease and 3 studies evaluating genus *Faecalibacterium* showed a significant decrease in IBS-D patients [21, 29–33]. In addition, the species-specific alterations of gut microbiota, such as *Bifidobacteria* and *Bacteroides*, were different between IBS patients from China and other regions [27], this discovery was similar with our study. A US study found that persons with a *Campylobacter* infection have a much higher risk of developing IBS compared with those not diagnosed with *Campylobacter* infection [34], which is consistent with our study. However, there were significant differences in the results of the studies on the changes of gut microbiota in IBS-D patients, which may be caused by regional and dietary factors.

### Predictive functional profiling of gut microbiota

There is ever-growing evidence supporting the role of microbes in the pathophysiology of IBS. Alterations in the gut microbiome may lead to impaired gut barrier function and potentially contribute to IBS symptoms [12]. *Firmicutes* makes up the largest portion of the human gut microbiota and has been shown to be involved in energy extraction and potentially related to obesity and diabetes [35]. *Bacteroidetes*, the second most abundant phylum in the human gut, generally produces butyrate, which is suggested to reduce inflammation and plays a role in the normal development of the gut [10]. *Proteobacteria* are one of the most abundant phyla, an increasing amount of data identifies it as a possible microbial signature of disease, and major pieces of evidence currently involve metabolic disorders and inflammatory bowel disease [36]. *Fusobacteria* is rare constituents of the fecal microbiota, but have been cultured previously from biopsies of inflamed gut mucosa [37]. In our study, decreased *Firmicutes, Fusobacteria* and increased *Proteobacteria* were found in IBS-D patients, which may be related to inflammation and metabolic disorder.

### Effect of staple food on gut microbiota and IBS-D

There are many factors affecting gut microbiota, such as dietary patterns (alcohol, caffeine, spicy food, elimination diets, fat and fluid intakes and dietary habits [38]), environment (temperature and humidity [39]) and pressure, etc. In this study, the influence of staple food on gut microbiota was observed.

China has undergone significant transitions in dietary patterns during the last several decades, one of the changes was that the shift of staple food consumption towards refined cereals (e.g., polished rice, white wheat) and away from traditional coarse staple foods (e.g., millet, sorghum) [40]. In China, people in different regions have different eating habits. The general staple food for people living south of Yangtze River is rice; in the area north of the Yangtze River, people subsist chiefly on wheat; whereas people in the north of the Yellow River, one of the major staple food is naked oats [41]. These differences led to the different incidence of IBS-D in China. An epidemiological study indicated that 277 (1.72%) had IBS-D in 16,078 respondents from 5 cities of China [42]. The incidence of IBS-D in Guangzhou and Wuhan were higher than that in Beijing [43–45]. A Japanese study reported that dietary interventions did not make any significant difference in the abundance of *Bacteroidetes* and *Firmicutes*, but the abundance of *Actinobacteria* was significantly increased after 7 days intake of white bread [46]. But in our study, at phylum level, there is no significant difference in abundance of gut microbiota between peoples who consume rice as a staple food (samples of A1, A5, A7, A10, B5, B7, B17) and peoples who consume wheat as a staple food. But the 7 samples have decreased *Firmicutes* (0.498 ± 0.179 vs. 0.61 ± 0.164, *P* = 0.09) and increased *Bacteroidetes* (0.299 ± 0.223 vs. 0.212 ± 0.167, *P* = 0.22) compared with other samples. Overall, these results indicated that effect of staple food on gut microbiota is not related to IBS-D.

The sample size of this study is small, so multi-center, large sample size and long-term clinical observation are still needed to confirm our results. In addition, there were significant differences in BMI between the two groups, which may be related to the selection of control population or gut microbiota, which still needs further study.

## Conclusion

According to our study, in Nanchang, China, IBS-D patients are characterized by complex and unstable gut microbiota compared with the normal population. Decreased *Firmicutes, Fusobacteria, Actinobacteria*, and increased *Proteobacteria* may be contributed to these differences by influencing inflammation and metabolism of host.

Gut microbiota composition is quite different between Chinese and Western IBS-D patients and the reasons need to be further studied. Effect of dietary patterns, such as staple food, on gut microbiota does not increase the incidence of IBS-D.

## Supplementary Information

**Additional file 1: Table S1.** Characteristic of two groups.

**Additional file 2: Table S2.** OTUs of two groups.

**Additional file 3: Table S3.** Class top10 of two groups.

**Additional file 4: Table S4.** Comparison of genus 35 clustering between two groups.

**Additional file 5: Table S5.** KEGG metagenome predictions.

**Additional file 6: Table S6.** Predicted metagenomes KEGG at L1.

**Additional file 7: Table S7.** Predicted metagenomes KEGG at L2.

**Additional file 8: Table S8.** Predicted metagenomes KEGG at L3.

## Data Availability

All available data and material are in the manuscript and Supplementary Material.

## Abbreviations

GI: Gastrointestinal
HCs: Healthy controls
IBD: Inflammatory bowel diseases
IBS-D: Diarrhea-predominant irritable bowel syndrome
KEGG: Kyoto Encyclopedia of Genes and Genomes
LDA: Linear discriminant analysis;
NMDS: Non-metric multi-dimensional scaling
OTUs: Operational taxonomic units
PCA: Principal co-ordinates
PCoA: Principal component analysis
PCR: Polymerase chain reaction
PICRUSt: Phylogenetic Investigation of Communities by Reconstruction of Unobserved States
UPGMA: Unweighted pair-group method with arithmetic mean
